# Metal Crack Length Prediction and Sensor Fault Self-Diagnosis Method Based on Deep Forest

**DOI:** 10.3390/s25237149

**Published:** 2025-11-23

**Authors:** Qiang Gao, Yang Meng, Hua Li, Bowen Yang, Junzhou Huo

**Affiliations:** 1 The Department of Mechanical Engineering, Dalian University of Technology, Dalian 116024, China; gao13qiang88@163.com (Q.G.); mengyang@mail.dlut.edu.cn (Y.M.); lihua0107@mail.dlut.edu.cn (H.L.); huojunzhou@dlut.edu.cn (J.H.); 2 School of Mechanical Engineering, Shenyang University of Technology, Shenyang 110870, China

**Keywords:** crack length prediction, multiple loads, strain compensation, deep forest, self-diagnosis

## Abstract

Metal structures develop cracks under fatigue loading, which subsequently propagate. The size of the cracks directly affects the fatigue life of the structure. Accurate prediction of crack lengths under various loading conditions is crucial for the safe service of structures. And the crack length has a significant influence on the local strain of the structure. In this paper, finite element analysis (FEA) is used to extract strain data from various measurement points of compressive and tensile (CT) specimens under different loading conditions. The Deep Forest (DF) model is employed to optimize the training of the data. Compensation is applied to the measured dynamic strain data for predicting crack length. Experimental results show that multi-dimensional input signals in the XY plane can accurately predict crack length. Additionally, based on the Pearson correlation coefficient, this paper proposes a self-diagnostic coefficient for strain sensors. Combined with the DF model, it enables self-diagnosis of the strain sensor. The proposed crack length prediction and strain sensor self-diagnosis methods enhance the intelligence level of crack state monitoring to some extent.

## 1. Introduction

Various loads will impact mechanical equipment in the service process. During long-term loading, stress concentration in key structural parts can lead to the formation of cracks [1]. The presence of structural cracks has a detrimental effect on the efficient and stable operation of the equipment [2,3]. In recent years, numerous efficient and accurate methods for monitoring damage have been developed [4]. Non-destructive monitoring technology can efficiently and accurately identify cracks without damaging the structural parts, making it the mainstream crack identification technology [5,6,7]. Various types of sensors can be utilized to monitor structural cracks, including piezoelectric [8,9], vision imaging [10], optical fiber [11], strain [12], and acoustic emission [13], vibration [14]. Various sensor monitoring methods have also been extensively studied by researchers.

The study of structural crack monitoring using nondestructive methods is also favored by researchers. Research has been conducted on crack identification using a piezoelectric sensor to actively stimulate Lamb waves. Qiu et al. predict multiple fatigue crack growth using a modified Paris model within a particle filtering framework [15]. Xu et al. utilized piezoelectric transducers to detect cracks in steel bars and investigated their effectiveness in identifying cracks under corrosive conditions [16]. Yuan et al. utilized a piezoelectric sensor in conjunction with a mixture proposal particle filter to perform online monitoring of double crack propagation [17]. Su et al. characterized fatigue crack growth using a model-driven approach [18]. With the advancement of ultrasonic technology, researchers have also explored the use of nonlinear ultrasonic signals for crack monitoring. Xiang et al. utilized the Lamb wave nonlinear ultrasonic method to monitor closed cracks in metals [19]. Cheng et al. monitor the cracks using a nonlinear ultrasonic phased array method [20]. There are also related studies on monitoring structural cracks using the acoustic emission method. Zhao et al. monitored structural fatigue cracks in CT samples using acoustic emission and strain sensors [21]. According to the crack characteristics of real engineering structures, there are also studies aimed at monitoring the cracks in these specific structures. He et al. monitored the crack growth on both sides of the center hole using strain sensors and predicted the crack using Gaussian process regression [22]. Shi et al. utilized multi-point strain monitoring to detect cracks at the threads of high-pressure pipelines and to identify the cracks by comparing the data with that from areas without cracks [23]. Li et al. conducted fatigue crack monitoring of large area bridge structures using wireless strain monitoring [24].Furthermore, cracks within the structure can also be identified through vibration signals. Qian et al. proposed a DG-Softmax model that can accurately detect cracks in planetary gears [25]. Certain scholars have researched novel sensor technologies tailored for the detection of structural cracks. Fan et al. have developed a ring sensor that can effectively monitor the propagation of fatigue cracks when installed in the crack region [26]. In recent years, with the development of machine learning technology, the application of machine learning methods to identify cracks has also made some progress. For example, machine learning and vision technology are used to monitor wall cracks [27]. And the structural cracks are identified by machine learning methods [28]. Cracks typically propagate to a certain length before causing structural failure [29]. Monitoring crack length also provides a valuable reference for the periodic maintenance of structures [30]. Accurate prediction of crack length can lay the foundation for understanding crack propagation patterns. Current crack detection methods are mostly limited to identifying and recognizing cracks, making it difficult to achieve accurate quantification of crack length. Vision-based deep learning crack detection methods are influenced by factors such as environmental conditions and camera field of view, which restrict their application scenarios. However, due to sensor limitations, most studies struggle to achieve accurate crack length prediction under dynamic loading conditions. Furthermore, current deep learning methods require large amounts of training data and continuous adjustment of optimization parameters to achieve optimal prediction performance. Currently, deep learning methods are mainly used for crack detection. However, the dynamic prediction of crack length still requires further research.

The Deep Forest (DF) model offers significant advantages for regression prediction with small sample data. Moreover, this model does not require manual optimization of network parameters, as it automatically optimizes the network structure parameters during training based on the complexity of the data [31]. The paper employs a deep forest model to bidirectionally train on a dataset extracted from FEA, resulting in two specialized models: **DF1** for predicting crack length and **DF2** for predicting strain state. The approach ultimately enables sensor fault diagnosis through a proposed self-diagnosis coefficient. The method presented in the paper achieves dynamic crack prediction in structures using only a small sample dataset. The second part of the paper introduces the crack prediction method and the framework for the sensor fault self-diagnosis principle. The third part describes the method for obtaining the FEA training dataset. The fourth part presents the experimental test system and the strain signal compensation method. The fifth part analyzes the model’s crack length prediction results and the self-diagnosis method’s outcomes. The sixth part summarizes the conclusions of this paper.

## 2. The Overall Framework of the Thesis

**Figure 1:**
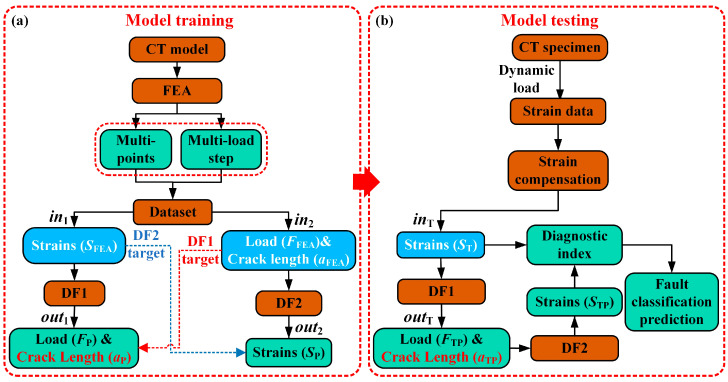
Model training and validation process. (**a**) Model training process, (**b**) model testing process.

**Figure 2:**
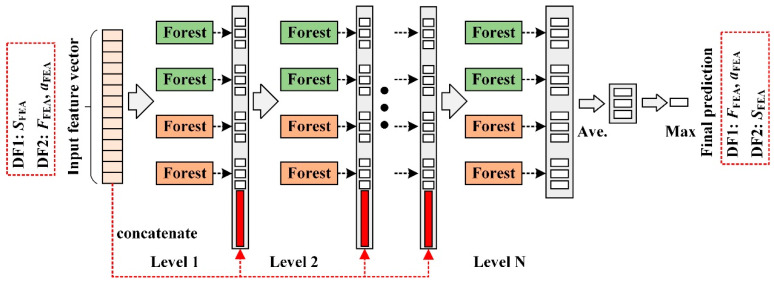
Theoretical framework of the deep forest model.

Figure 1 shows the flowchart of model training and testing. In Figure 1a, *S*_FEA_ represents the strain data extracted from FEA, while *F*_FEA_ and *a*_FEA_ denote the load and crack length from the FEA dataset, respectively. Together, these constitute the training dataset for the models. The input *in*_1_ to the **DF1** model is *S*_FEA_, and its output *out*_1_ consists of the predicted load *F*_P_ and predicted crack length *a*_P_. The training targets for the **DF1** model are *F*_FEA_ and *a*_FEA_ from the dataset. For the **DF2** model, the input *in*_2_ is *F*_FEA_ and *a*_FEA_, and its output *out*_2_ is the predicted strain *S*_P_. In Figure 1b, the strain data from the CT specimen under dynamic loading first undergoes strain compensation to generate the test input *in*_T_ for the model. *S*_T_ represents the tested strain data, which is fed into the trained **DF1** model. The model output *out*_T_ includes the predicted load *F*_TP_ and crack length *a*_TP_. *F*_TP_ and *a*_TP_ then serve as the input to the **DF2** model, producing the output strain *S*_TP_. Finally, by comparing *S*_T_ and *S*_TP_, the damage index is used to predict the type of sensor fault. The DF model performs feature extraction through multiple forest modules and achieves final prediction through multi-level feature fusion, as shown in Figure 2.

## 3. Dataset Construction

**Figure 3:**
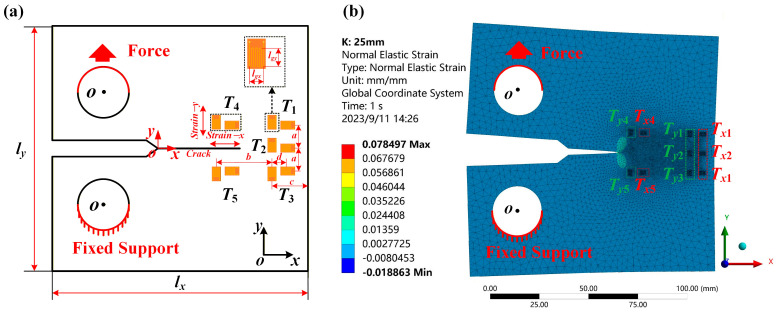
Schematic diagram of CT specimen and FEA strain test area. (**a**) Strain gauge position diagram, (**b**) schematic diagram of the strain test area for FEA.

Figure 3 depicts the FEA structural model and the simulated strain cloud image. Figure 3a displays the dimensions of structural components utilized in FEA and the position of the strain gauge. The parameters of each structural model are shown in Table 1. The ANSYS Workbench 2021 statics module is used to simulate the strain of CT sample models with different crack lengths. In this paper, 4330 V steel is used as the test material, and its material parameters are shown in Table 2. The material parameters have been set, and the structure has been meshed. The strain measuring points are divided into corresponding rectangular areas before model simulation. For the 10 measuring points in the CT sample, the rectangular strain regions are divided based on the test direction and the position of the strain gauge. To enhance the accuracy of the simulated strain, the mesh of the strain monitoring region is subdivided. A tetrahedral mesh is utilized, with a mesh size set to 2 mm in the non-strain monitoring area. The minimum mesh size in the strain monitoring area is set to 0.2 mm. After meshing, the load parameters for the simulation structure model are set. In this paper, the load parameters are defined during the load step. In this study, the CT specimen was loaded in 20 steps, with the load increasing from 2 kN to 40 kN in increments of 2 kN. The crack length was simulated from 5 mm to 55 mm, using increments of 2 mm. This process generated a total of 520 FEA dataset samples. After solving, the strain along the *x*-axis and *y*-axis at different measuring points *T*_1_–*T*_5_ is extracted, respectively. In order to enhance the accuracy of the simulation strain, the paper takes the average value of the strain of each strain test area node as the final simulation strain values.

## 4. Experimental Measurement

### 4.1. Test System Construction

**Figure 4:**
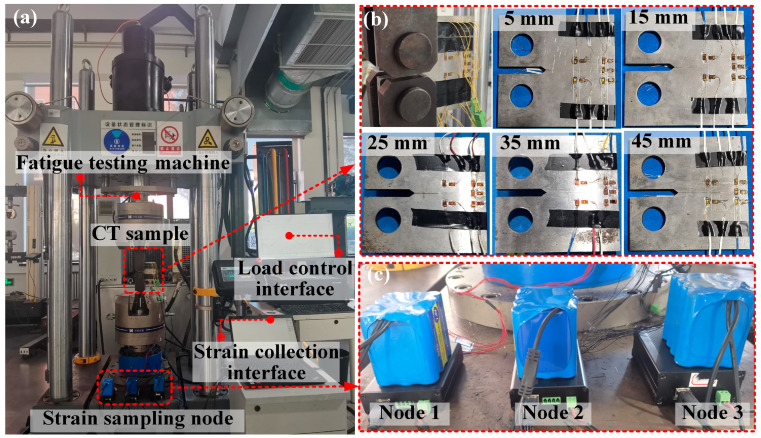
Multiple load experimental system. (**a**) Experimental testing system, (**b**) CT specimens, (**c**) strain signal sampling node.

Figure 4 shows a multiple-load experimental system for CT sample testing. The fatigue testing machine is controlled through the load control interface, allowing for the application of both static and dynamic loads to the CT sample. The strain at each measurement point on the CT specimen is monitored through strain sampling nodes. The strain acquisition unit used in this study is a self-developed strain acquisition node [32]. The strain sampling rate was set to 25 Hz, while the frequency of the dynamic loading was 1 Hz. The CT specimens in Figure 4b are tested by the testing machine under static and dynamic loads. The parameters for model training are shown in Table 3.

The method proposed in the paper achieves crack prediction with a small sample dataset. Model training was conducted on a CPU (Intel i5-10400 @ 2.9 GHz, 12 cores) with 32 GB of RAM, requiring 3.7339 s to complete using 520 training samples.

### 4.2. Strain Data Analysis Under Static Load

**Figure 5:**
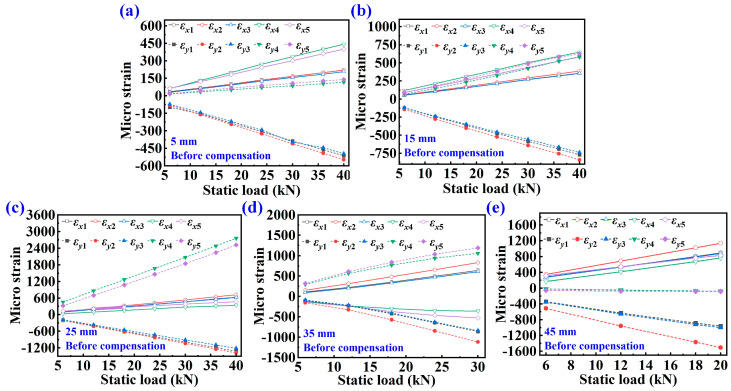
Strain data at each measuring point under static load before compensation. (**a**) 5 mm crack length, (**b**) 15 mm crack length, (**c**) 25 mm crack length, (**d**) 35 mm crack length, (**e**) 45 mm crack length.

Figure 5 illustrates the strain of various CT samples under different static loads. Figure 5a–e shows the monitored strain with crack lengths of 5 mm, 15 mm, 25 mm, 35 mm, and 45 mm, respectively. The tensile and compressive stresses at each measuring point are different under the action of a static load. The strain is positive when subjected to tensile stress and negative when subjected to compressive stress. As the static loading force increases, the tensile and compressive stress at each measuring point also increase linearly. The tensile or compressive stress at each measuring point will also change as the crack length changes.

Furthermore, the variation in monitored strain differs at various measuring points due to differences in the installation positions of the strain gauges. Due to the non-uniformity of material properties, the monitored strain and simulation results of the measuring point under the same load will also differ. This paper compares the measured strain to the strain value obtained from FEA. Formula (1) demonstrates that the actual force $F_{E}$ is linearly related to the structural stress $\sigma_{E}$ in elastic deformation. And the structural stress $\sigma_{E}$ is also linearly related to the strain $\varepsilon_{E}$. Similarly, the simulated force $F_{F}$, stress $\sigma_{F}$ and strain $\varepsilon_{F}$ in FEA are also linearly correlated, as shown in Formula (2). The FEA force $F_{F}$ is assumed to be equivalent to the actual force $F_{E}$. Therefore, the monitored strain $\varepsilon_{E}$ and the simulated strain $\varepsilon_{F}$ are also linearly correlated, as shown in Formula (3).

(1) $$F_{E}\propto \sigma_{E}\propto \varepsilon_{E}$$

(2) $$F_{F}\propto \sigma_{F}\propto \varepsilon_{F}$$

(3) $$\sigma_{E}\propto \sigma_{F}$$

The parameter matrices ***A*** and ***B*** are obtained by linear fitting analysis between the monitor strain data and the corresponding FEA strain data, as shown in Formulas (4) and (5) where *n* is a positive integer.

(4) $$A=\left[\begin{array}{cccc} a_{1} & a_{2} & \ldots & a_{n} \end{array}\right]$$

(5) $$B=\left[\begin{array}{cccc} b_{1} & b_{2} & \ldots & b_{n} \end{array}\right]$$

According to the parameter matrix of different measuring points, the strain $\varepsilon_{P}$ with error compensation is calculated, as shown in Formula (6).

(6) $$\varepsilon_{P}=A\varepsilon_{P}+B$$

The parameter matrices ***A*** and ***B***, including error compensation, are shown in Table 4. In the table, the strain compensation parameter values for the corresponding 5 mm–45 mm specimens of ***A*** and ***B*** are represented by *A*_1_–*A*_5_ and *B*_1_–*B*_5_ respectively. *x*_1_–*x*_5_ and *y*_1_–*y*_5_ represent the strain compensation values of the five test areas of the CT specimens along the *x*-axis and *y*-axis. The magnitude of the *A* value and the *B* value represents the degree of deviation between the actual test strain and the compensated strain. The larger the deviation of the *A* value from 1, and the larger the absolute value of the *B* value, the greater the deviation between the actual test strain and the theoretical strain.

**Figure 6:**
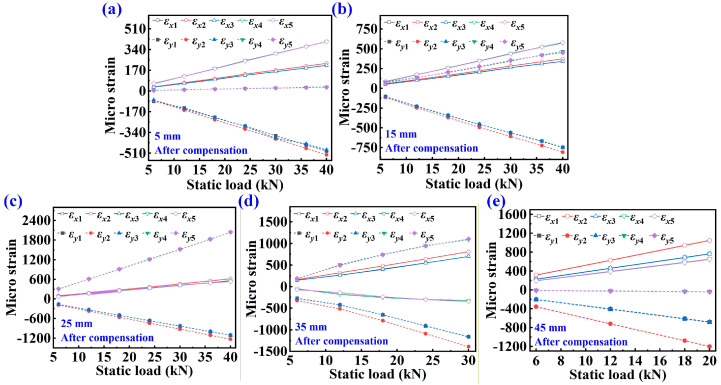
Strain with strain error compensation under static load after compensation. (**a**) 5 mm crack length, (**b**) 15 mm crack length, (**c**) 25 mm crack length, (**d**) 35 mm crack length, (**e**) 45 mm crack length.

The strain data with error compensation under static load is shown in Figure 6. It can be seen from the figure that the strain and load still have a linear relationship after error compensation. Because the measuring points on both sides of the crack are arranged symmetrically, the strain data of the measuring points *T*_1_ and *T*_3_, *T*_4_ and *T*_5_ along the *x* and *y* axes are theoretically the same. After error compensation, the strain data for *T*_1_ and *T*_3_, *T*_4_, and *T*_5_ of different CT specimens are essentially consistent.

### 4.3. Test Data Analysis Under Dynamic Load

**Figure 7:**
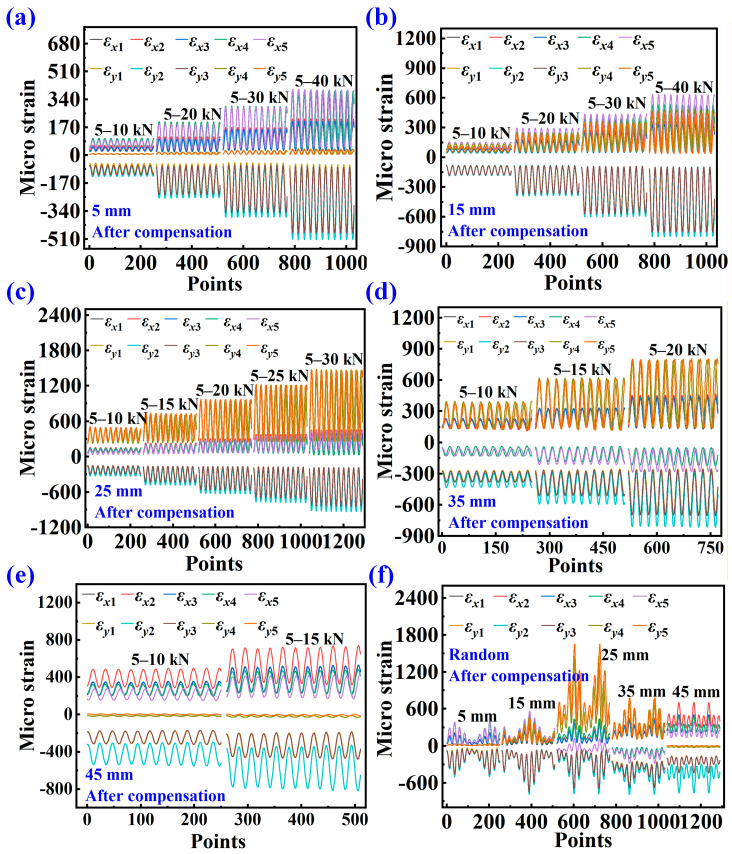
Strain monitoring results of CT samples with various cracks under different cycle loads and random loads. (**a**) 5 mm crack length, (**b**) 15 mm crack length, (**c**) 25 mm crack length, (**d**) 35 mm crack length, (**e**) 45 mm crack length, (**f**) the strains of CT specimens with different crack lengths under random loads.

Figure 7 displays the strain monitoring results for *T*_1_–*T*_5_ along the *x* and *y* directions under various cycle loads. The *ε_x_*_1_–*ε_x_*_5_ represent the strain along the *x*-axis, while the *ε_y_*_1_–*ε_y_*_5_ represent the strain along the *y*-axis. The cycle load follows the standard sine function, and the setting parameters are presented in Table 5. The cyclic load is shown by the Formula (7).

(7) $$F_{D}=F_{a}\sin2\pi ft+F_{m}$$

where *F_a_* represents the amplitude of the cycle load, and *F*_m_ represents the mean value of the cycle load. In Table 5, *F*_min_ and *F*_max_ represent the minimum and maximum values of the load, respectively.

Figure 7a–e shows the dynamic strain under a single-cycle load. Under the action of the cycle load, the dynamic strain at each measuring point also exhibits periodic changes. The tension and compression states result in varying strain changes at different measuring points. The absolute value of strain at different measuring points changes noticeably with the variation in crack length. As the cycle load increases, the strain value at each measuring point also increases. Figure 7f displays the monitoring results of each strain measuring point under random loads. The parameters are shown in Table 6. The random load consists of several single-cycle sinusoidal loads, as shown in Figure 7. During the random loading process, the strain variation follows the same pattern as the random load.

## 5. Analysis of Experimental Results

### 5.1. Analysis of Crack Prediction Results

**Figure 8:**
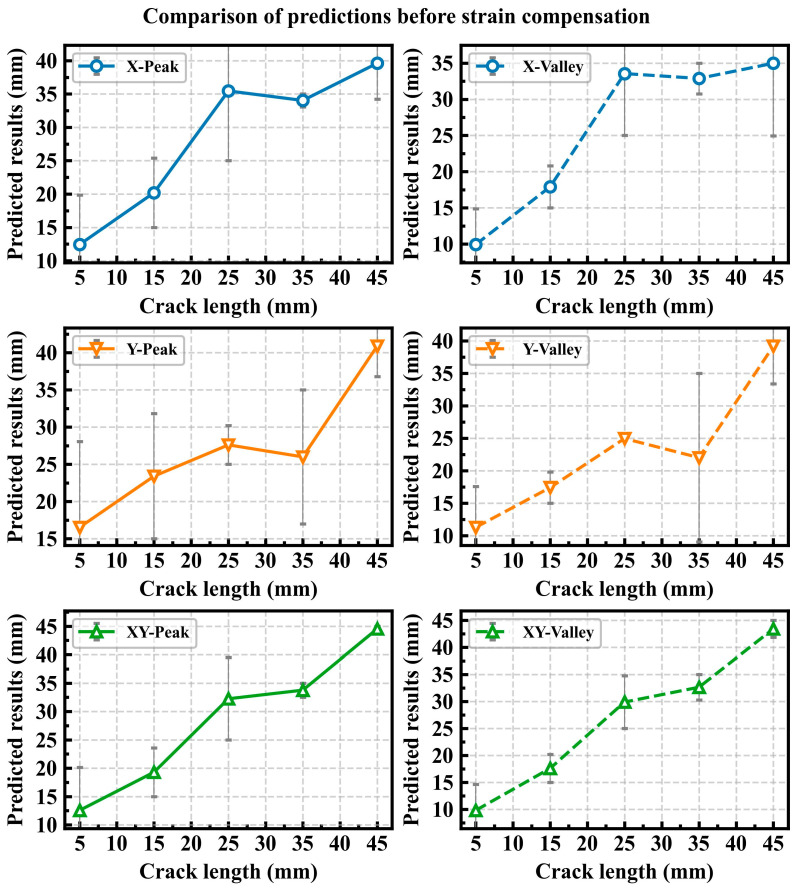
Comparison of crack length prediction results under different strain input conditions before strain compensation.

**Figure 9:**
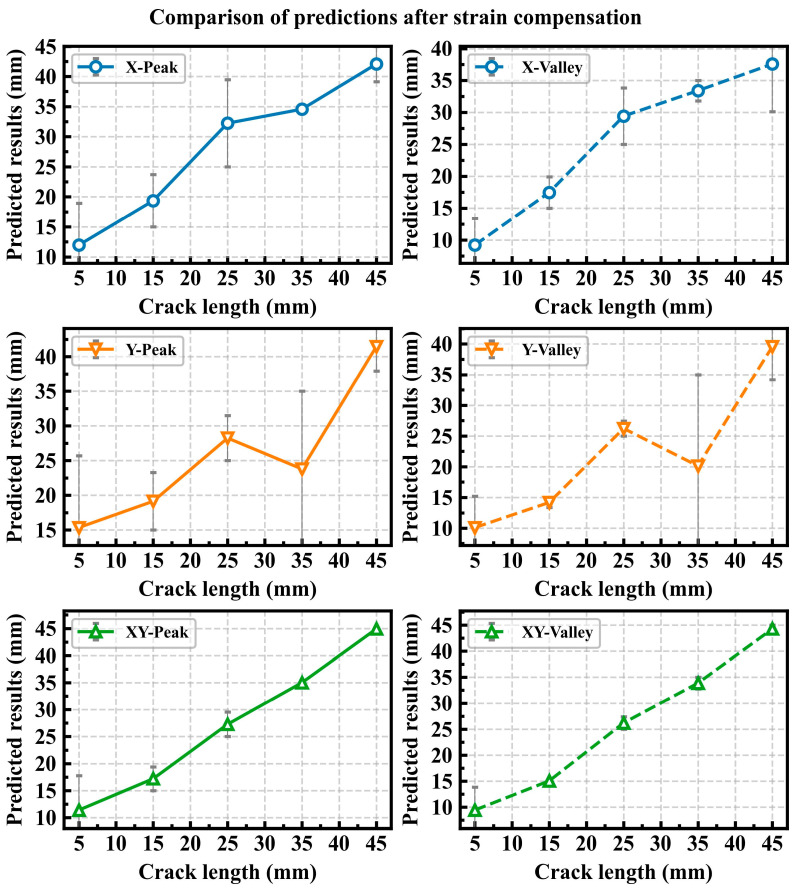
Comparison of crack length prediction results under different input conditions after strain compensation.

Figure 8 shows the error bar chart of crack length prediction results under different strain input conditions before strain compensation. The input strain conditions are mainly divided into three types: X represents the input from the *x*-axis strain alone, Y represents the input from the *y*-axis strain alone, and XY represents the input from all strain channels in both the *x* and *y* axes. Peak represents the peak value of the dynamic strain, while Valley represents the valley value of the dynamic strain. For example, X-Peak represents the peak value of the dynamic strain along the *x*-axis of the input; X-Valley represents the valley value of the dynamic strain along the *x*-axis of the input. The chart compares the prediction results for CT specimens with crack lengths of 5 mm, 15 mm, 25 mm, 35 mm, and 45 mm. Figure 9 shows the error bar chart of prediction results under different input conditions after strain compensation. From

Figure 8 and Figure 9, it can be observed that the prediction error under the XY input condition is smaller than that under the X and Y strain input conditions.

Table 7 and Table 8 present a comparison of crack length prediction relative percent errors (RPEs) before and after strain compensation, respectively. The RPE calculation method is shown in the Formula (8).

(8) $$\mathrm{RPE}=\left|\frac{{\hat{y}}_{i}-y_{i}}{y_{i}}\right|\times 100$$

where ${\hat{y}}_{i}$ represents the predicted value, $y_{i}$ represents the actual value.

The data in the tables indicate that the RPE decreases as the crack length increases. When the crack length exceeds 25 mm, the RPE after strain compensation is generally maintained within 15%. Moreover, when the input utilizes XY strain data, the RPE remains largely within 10%. Table 9 demonstrates the percentage reduction in RPE after strain compensation compared to that before compensation. The data in the table show that strain compensation effectively reduces the RPE in the majority of cases. The reduction is most pronounced when XY strain data are used as input. For crack lengths beyond 15 mm, the RPEs for both XY-Peak and XY-Valley are reduced by over 50%. The comparison of data in the tables confirms the importance of both the *x*-axis and the *y*-axis strain for accurate crack prediction. Uniaxial strain alone is insufficient for accurately predicting structural crack length, while the combination of *x*-axis and *y*-axis strain provides multi-dimensional signal characteristics that enhance prediction accuracy. Different magnitudes of dynamic loads result in varying crack propagation rates; the higher the dynamic load, the faster the crack grows, and vice versa. Comparing the prediction errors at peak and valley loads is intended to establish the maximum and minimum ranges of prediction error. The prediction error is greatest when the load is at its minimum and smallest when the load is at its maximum. In practice, the strain under the peak value of alternating load is typically selected as the model input to predict crack length.

When the crack length is 5 mm, the prediction errors are significantly large, and in some cases even exceed 100%. This occurs because, with small cracks, the strain values at each measurement point remain very low even under relatively high loads. Low strain values amplify the discrepancy between the measured and FEA strain results. This issue persists even after strain compensation, ultimately leading to increased prediction errors for small crack lengths.

**Figure 10:**
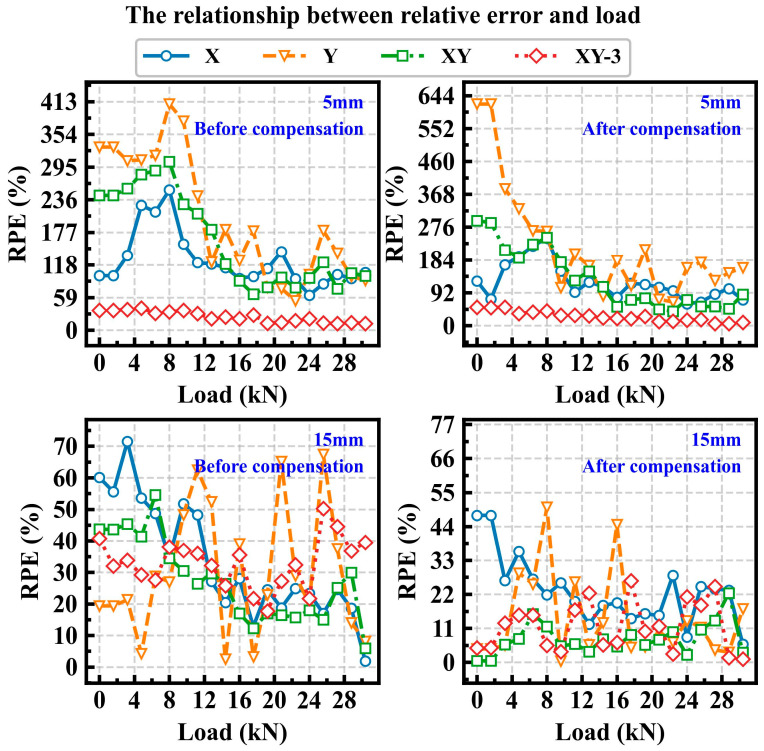
The relationship between the RPE and the load under crack lengths of 5 mm and 15 mm.

**Figure 11:**
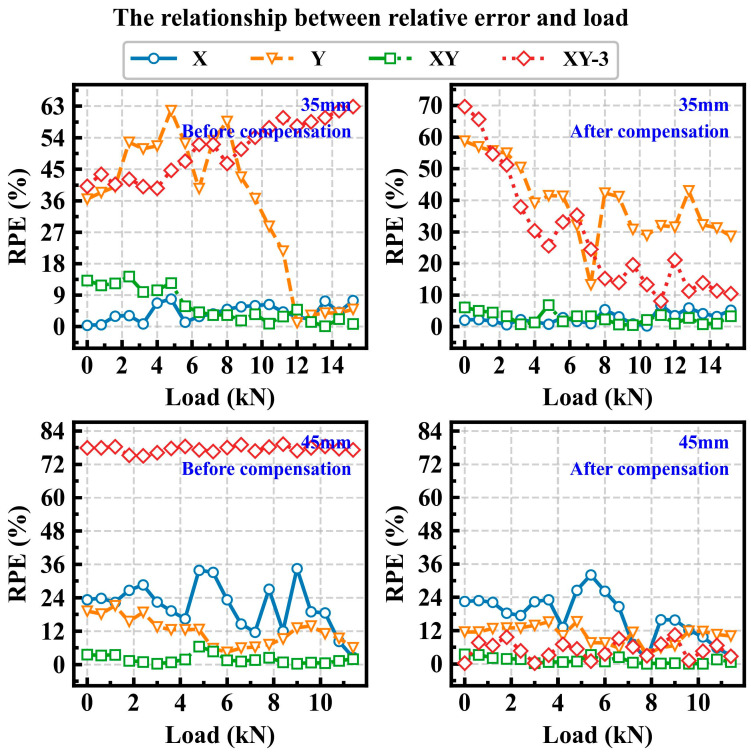
The relationship between the RPE and the load under crack lengths of 35 mm and 45 mm.

Figure 10 and Figure 11 illustrate the relationship between RPE and load under different crack lengths. As shown in Figure 10, for crack lengths of 5 mm and 15 mm, the RPE gradually decreases as the load increases. This phenomenon is more pronounced for X and XY input conditions. Furthermore, after compensation for the strain data, the prediction error is also reduced. As shown in Figure 11, when the crack length increases to 35 mm or more, the RPE of the XY remains relatively low. And under the XY input condition, for a crack length of 35 mm, the RPE can be maintained within 10% across all load states. Moreover, when the crack grows to 45 mm, the RPE remains within 5% across the entire load range. Here, XY-3 represents the input condition where the strain data consists of *x*_1_–*x*_3_ and *y*_1_–*y*_3_. Analysis of Figure 10 and Figure 11 indicates that the prediction performance under the XY-3 input condition is suboptimal. Therefore, based on the analysis of the prediction results, it can be concluded that the contributions of the *x*-axis and *y*-axis strain from each measurement point vary across different stages of crack growth, and the XY input condition yields the best prediction accuracy for all crack lengths.

### 5.2. Self-Diagnosis of Strain Sensors

The mean absolute percentage error (MAPE) reflects the deviation between predicted results and actual results, as shown in Formula (9).

(9) $$\mathrm{MAPE}=\frac{100\%}{n}\sum_{i=0}^{n}\left|\frac{{\hat{y}}_{i}-y_{i}}{y_{i}}\right|$$

**Figure 12:**
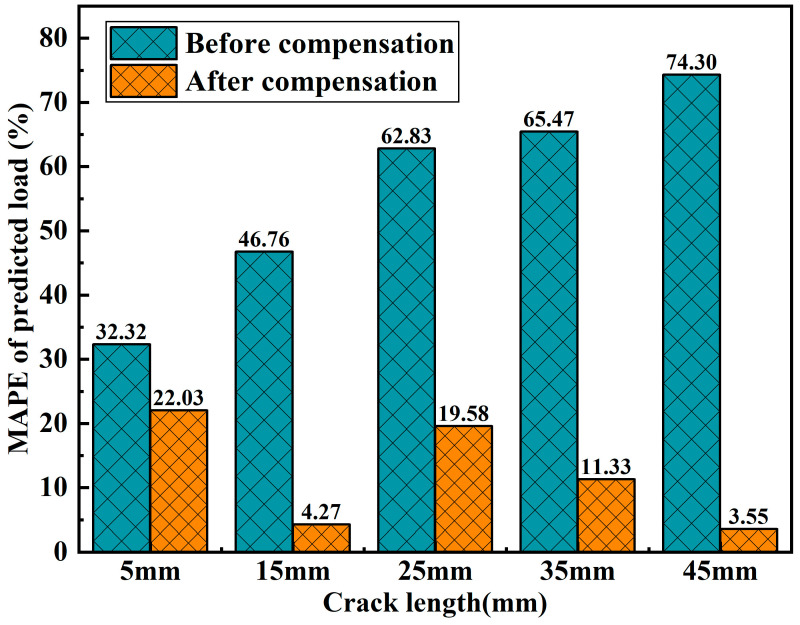
A comparison of MAPE for predicted load under XY input conditions, before and after strain compensation.

In the formula, ${\hat{y}}_{i}$ represents the predicted value, $y_{i}$ represents the actual value, and *n* represents the number of samples involved in the calculation. The bar chart in Figure 12 displays the MAPE of the load predicted by the **DF1** model under different crack lengths, both before and after strain compensation. It can be clearly observed from the figure that the load prediction error for the XY case is significantly reduced after strain compensation. Moreover, as the crack length increases, the MAPE of the load prediction results gradually decreases after strain compensation. Compared to the uncompensated case, the reduction in MAPE for load prediction is smallest at a crack length of 5 mm, with a decrease of 31.84%. At a crack length of 45 mm, the percentage reduction in MAPE is largest, reaching 95.22%. The accuracy of load prediction directly determines the performance of the sensor’s self-diagnostic fault prediction results.

**Figure 13:**
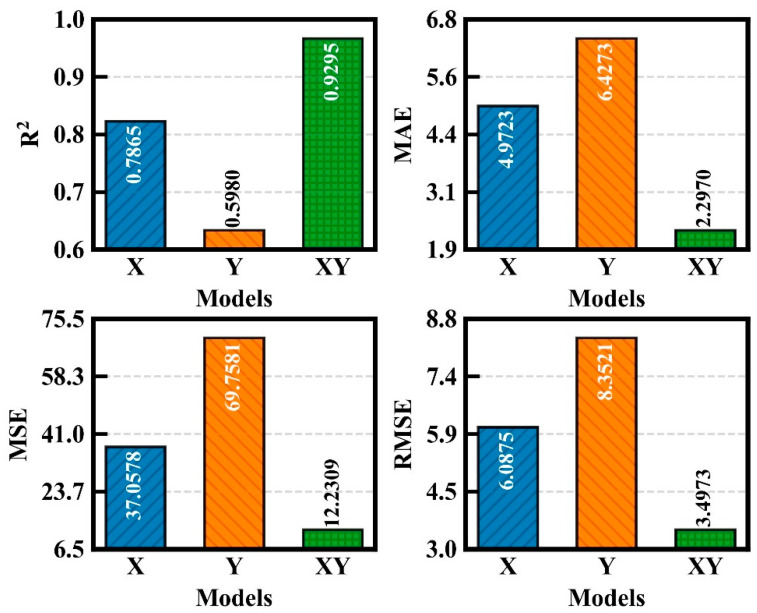
Comparison of each evaluation index after compensation for variations.

The paper conducted a statistical analysis of the prediction results, including the determination coefficient (R^2^), mean absolute error (MAE), mean square error (MSE) and root mean square error (RMSE), for all CT specimens under different loading conditions. These metrics were evaluated using a total of 2300 test samples. The results under X, Y, and XY inputs were compared, as shown in Figure 13. The comparison demonstrates that the XY case achieved the highest R^2^ value of 0.9295, while also yielding the relatively lowest MAE, MSE, and RMSE values of 0.2970, 12.2309, and 3.4973, respectively.

Table 10 and Table 11 present the improvement in prediction performance achieved by using XY compared to X and Y, before and after strain compensation, respectively. Here, XY_X denotes the improvement of XY over X, and XY_Y represents the improvement of XY over Y. The data in both tables indicate that, both before and after strain compensation, the XY condition consistently yields better prediction performance than either X or Y alone. This also confirms that utilizing XY effectively enhances the model’s prediction accuracy. The calculation methods are shown in Formulas (10)–(13).

(10) $$R^{2}=1-\frac{\sum_{i=1}^{n}{\left(y_{i}-{\hat{y}}_{i}\right)}^{2}}{\sum_{i=1}^{n}{\left(y_{i}-\bar{y}\right)}^{2}}$$

(11) $$MSE=\frac{1}{n}\sum_{i=1}^{n}{\left(y_{i}-{\hat{y}}_{i}\right)}^{2}$$

(12) $$RMSE=\sqrt{\frac{1}{n}\sum_{i=1}^{n}{\left(y_{i}-{\hat{y}}_{i}\right)}^{2}}$$

(13) $$MAE=\frac{1}{n}\sum_{i=1}^{n}|y_{i}-{\hat{y}}_{i}|$$

where $y_{i}$ represents the true value, ${\hat{y}}_{i}$ represents the predicted value, and $\bar{y}$ represents the mean of the true values.

To ensure the persuasiveness of the comparison results, Table 12 presents the changes in various statistical indicators after strain compensation relative to those before compensation. Positive values in the table indicate improvement in the indicators, while negative values indicate deterioration. The data in the table demonstrate that, regardless of whether the input condition is X, Y, or XY, the prediction results improve after strain compensation. For the XY input condition, the R^2^ value increases by 18.94%, while the MAE, MSE, and RMSE decrease by 53.94%, 67.74%, and 43.2%, respectively. The data in the table validate the effectiveness of strain compensation in improving prediction performance.

In practical applications, strain gauges are inevitably susceptible to certain degrees of damage, such as degumming or fractures. This necessitates fault diagnosis for strain sensors to guide their replacement, thereby promptly preventing increased errors in crack length predictions caused by sensor failures during testing. If the dynamic strain sequences from the normal service condition of strain sensors are directly used as reference values for damage calculation, the strain sequences under normal service conditions for multiple channels would need to be recorded. This would require building a database of standard strain sequences and comparing measured data sequences with these standard sequences to identify the fault or damage state of the strain sensors. However, such an approach would complicate the entire diagnostic process and reduce efficiency.

In this paper, the crack length and load are used as input conditions, while the strain values of each channel serve as the output to train the DF model, resulting in model **DF2**. **DF2** can predict the current strain values of each channel based on the existing crack length and load conditions. Building on the Pearson correlation coefficient, this paper proposes a self-diagnostic coefficient $C_{d}$ for strain sensors, as shown in the Equation (14) below. The fault determination of the strain sensor is achieved by comparing the self-diagnosis coefficient of the predicted strain by **DF2** with the value of the actual dynamic strain data.

(14) $$C_{d}=r\times p$$

where *r* represents the Pearson correlation coefficient, and its calculation method is shown in Formula (15).

(15) $$r=\frac{\sum_{i=1}^{n}\left(x_{i}-x_{m})(y_{i}-y_{m}\right)}{\sqrt{\sum_{i=1}^{n}{\left(x_{i}-x_{m}\right)}^{2}\sum {\left(y_{i}-y_{m}\right)}^{2}}}$$

In the formula, ***x*** represents the actual measured strain sensor data sequence, ***y*** denotes the strain data predicted by the **DF2** model, and *x*_m_ and *y*_m_ represent the mean values of the measured and predicted strain data, respectively. The symbol *p* denotes the proportionality coefficient, as shown in Formula (16).

(16) $$p=\left(1-\frac{\left|x_{m}-y_{m}\right|}{\left|x_{m}+y_{m}\right|}\right)\cdot \frac{\left|x_{\max}-x_{\min}\right|}{\left|y_{\max}-x_{\min}\right|}$$

In the formula, *x*_max_ and *x*_min_ represent the maximum and minimum values of the measured strain sequence, respectively, while *y*_max_ and *y*_min_ represent the maximum and minimum values of the strain sequence predicted by the **DF2**.

During actual testing, if a strain sensor experiences debonding or aging failure, the actual measured strain value will significantly decrease under the same load conditions. In this study, 0.2 times the actual strain value is used to simulate strain data from a debonded strain gauge, and setting a specific channel’s strain to a constant value simulates a fractured strain gauge. To evaluate the effectiveness of strain sensor damage diagnosis, two channels of each tested CT specimen were configured with faults, with specific faulty channel settings detailed in Table 13.

**Figure 14:**
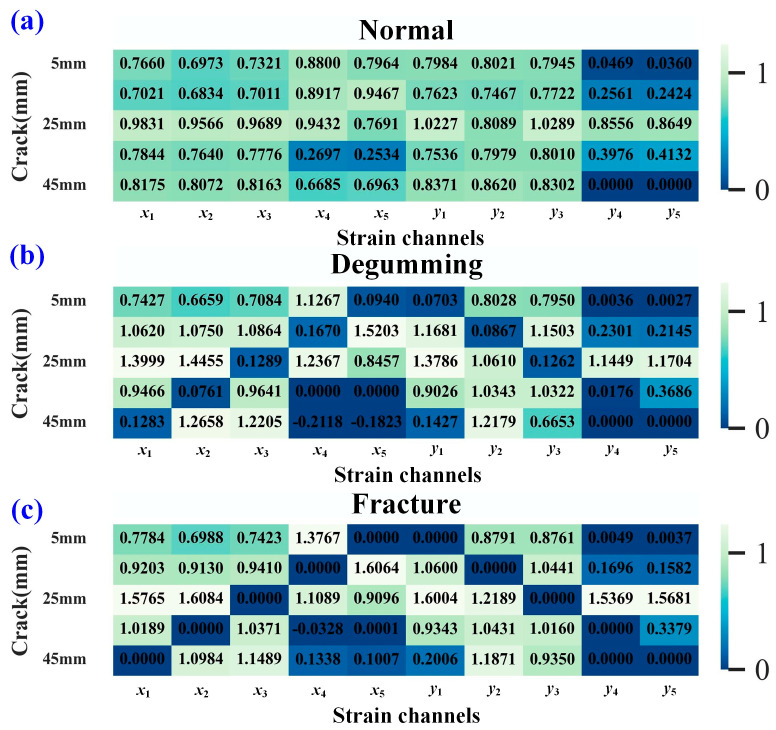
Diagnosis results of strain damage for each channel under XY input conditions. (**a**) normal condition, (**b**) degumming situation, (**c**) fracture situation.

Figure 14 shows the diagnostic results of the strain fault index under the XY input condition. As seen in Figure 14a, under normal sensor conditions, the strain fault indices for channels *y*_4_ and *y*_5_ are relatively small, resulting in false detections with a misdiagnosis rate of 20%. This occurs because the strain values of channels *y*_4_ and *y*_5_ are generally small and minimally influenced by load. The misdiagnosis rate for degumming conditions is 24%, while for fracture conditions, it is 4%.

**Figure 15:**
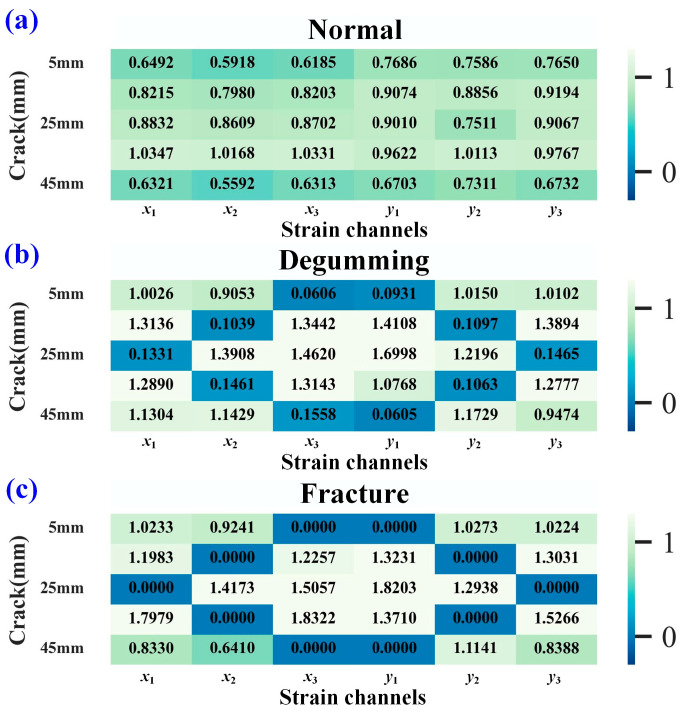
Diagnosis results of strain damage for each channel under XY-3 input conditions. (**a**) normal condition, (**b**) degumming situation, (**c**) fracture situation.

Figure 15 presents the calculated strain fault indices for each sensor under the XY-3 input condition. Figure 15a shows the results under normal sensor conditions, while Figure 15b displays the results under degumming conditions. Analysis of these figures indicates that the damage index values for degumming sensors are relatively small, allowing clear differentiation of their faulty state, which is represented by darker colors in the heatmap. When a strain sensor fractures, the calculated damage index drops to 0, as shown in Figure 15c. Under the XY-3 input condition, the fault diagnosis accuracy reaches 100%, with a misdiagnosis rate of 0%. These diagnostic results demonstrate that the dimensionality of the input strain data also influences the damage diagnosis outcome to some extent. Higher input strain dimensionality leads to a higher misdiagnosis rate. In practical monitoring, the XY input condition is used for crack length prediction, while the XY-3 condition can be combined with it for cross-validating strain sensor faults. Based on the self-diagnosis results of the predictive sensors, the diagnostic accuracy of XY-3 is higher than that of XY. This is because the **DF2** model takes both crack length and load as inputs and outputs strain values from multiple measurement points. When the number of output variables is excessively large, the predictive accuracy of the model tends to decrease.

## 6. Conclusions

The paper utilizes strain data extracted from FEA to train the **DF1** and **DF2** models, achieving crack length prediction and sensor fault self-diagnosis with small sample data. Experimental results demonstrate that the XY input condition yields smaller prediction errors compared to uniaxial strain input conditions X and Y, enabling accurate crack length prediction. As the load and crack length increase, the prediction error of the XY model decreases. When the crack length exceeds 35 mm, the prediction error remains within 10% across all load conditions. Furthermore, when the crack length reaches 45 mm, the prediction error stays within 5%. The proposed strain sensor self-diagnosis method, under XY input conditions, shows a false detection rate of 20% for normal sensors, 24% for degumming faults, and 4% for fracture faults. However, with XY-3 input conditions, it achieves 100% accuracy in classifying and diagnosing sensor fault types with a 0% misdiagnosis rate.

The proposed method not only enables accurate crack length prediction with limited sample data but also adapts to various loading conditions. Simultaneously, it can also achieve self-diagnosis of the faults of strain sensors that are pasted using the paste method, further promoting the intelligent application of crack condition monitoring. The method proposed in this paper is applicable to single-axis complex loading conditions and isotropic materials. In future research, we will conduct studies on the prediction methods for multi-axis loads and small cracks and fatigue life.

## Figures and Tables

**Table 1:** The structural model parameters. (Unit: mm).

Parameters	*l_x_*	*l_y_*	*a*	*b*	*c*	*d*	*l_gx_*	*l_gy_*
Values	125	120	10	30	12.5	7.5	3	2

**Table 2:** The simulation parameters of the 4330 V material.

Parameters	Density (kg/m^3^)	Young’s Modulus (GPa)	Poisson’s Ratio
Values	7850	206	0.3

**Table 3:** The basic parameters for the model’s training.

Parameters	Stop Threshold	Trees	Layers
Values	1 × 10^−6^	200	2

**Table 4:** Strain compensation parameters for each measuring point.

Crack (mm)	5		15		25		35		45	
*A*, *B*	*A*_1_	*B*_1_	*A*_2_	*B*_2_	*A*_3_	*B*_3_	*A*_4_	*B*_4_	*A*_5_	*B*_5_
*x*_1_	0.97	−3.24	0.95	2.03	0.88	1.38	1.03	38.01	0.88	−15.47
*x*_2_	1.01	3.23	0.97	0.28	0.86	−0.27	0.95	21.24	0.93	−8.39
*x*_3_	1.02	0.01	0.97	1.13	0.89	−1.12	1.07	52.86	0.89	−14.76
*x*_4_	0.91	1.88	0.93	−26.51	1.48	29.82	1.10	80.21	0.76	68.14
*x*_5_	1.03	−4.05	1.00	−3.18	1.38	−110.97	0.71	29.71	0.88	−83.47
*y*_1_	0.98	12.56	0.99	17.92	0.84	4.56	1.17	−155.40	0.77	64.24
*y*_2_	0.95	−3.73	1.00	31.01	0.90	3.14	1.11	−148.88	0.85	84.43
*y*_3_	0.96	−5.83	1.04	21.92	0.92	5.46	1.18	−166.29	0.74	65.85
*y*_4_	0.28	0.53	0.75	26.52	0.75	−40.24	1.19	−168.52	0.45	−3.06
*y*_5_	0.21	0.26	0.71	5.77	0.79	59.28	1.05	−148.44	0.94	40.37

**Table 5:** The cycle load setting parameter. (Unit: kN).

*F*_min_–*F*_max_	5–10	5–15	5–20	5–25	5–30	5–40
*F_a_*	2.5	5	7.5	10	12.5	17.5
*F*_m_	7.5	10	12.5	15	17.5	22.5

**Table 6:** The random load parameters *F*_min_–*F*_max_ for a single cycle. (unit: kN).

Crack Length (mm)	Cycle 1	Cycle 2	Cycle 3	Cycle 3	Cycle 4	Cycle 5	Cycle 6	Cycle 7	Cycle 8
5	5–10	5–20	5–30	5–40	5–30	5–20	5–10	N/A	N/A
15	5–10	5–20	5–30	5–40	5–30	5–20	5–10	N/A	N/A
25	5–10	5–15	5–20	5–25	5–30	5–25	5–20	5–15	5–10
35	5–10	5–15	5–20	5–15	5–10	N/A	N/A	N/A	N/A
45	5–10	5–15	5–10	N/A	N/A	N/A	N/A	N/A	N/A

**Table 7:** Comparison of RPEs before compensation. (Unit: %).

Crack (mm)	X-Peak	Y-Peak	XY-Peak	X-Valley	Y-Valley	XY-Valley
5	148.68	230.71	151.25	98.52	125.78	96.48
15	34.68	56.04	28.64	19.39	16.07	17.37
25	41.83	10.44	29.01	34.2	0.17	19.58
35	2.77	25.72	3.52	6.06	37.09	6.74
45	11.98	9.14	1.02	22.27	12.89	3.52

**Table 8:** Comparison of RPEs after compensation. (Unit: %).

Crack (mm)	X-Peak	Y-Peak	XY-Peak	X-Valley	Y-Valley	XY-Valley
5	139.3	206.83	127.75	83.98	102.05	88.75
15	29.05	27.55	14.64	16.38	5.42	0.39
25	28.93	12.97	9.09	17.62	4.9	4.78
35	1.26	32.05	0.02	4.61	42.52	3.21
45	6.54	7.85	0.16	16.56	12.04	1.63

**Table 9:** Compared to the results before strain compensation, the percentage reduction in RPE of the predicted results after strain compensation. (Unit: %).

Crack (mm)	X-Peak	Y-Peak	XY-Peak	X-Valley	Y-Valley	XY-Valley
5	−6.31	−10.35	−15.54	−14.76	−18.87	−8.01
15	−16.23	−50.84	−48.88	−15.52	−66.27	−97.75
25	−30.84	24.23	−68.67	−48.48	2782.35	−75.59
35	−54.51	24.61	−99.43	−23.93	14.64	−52.37
45	−45.41	−14.11	−84.31	−25.64	−6.59	−53.69

**Table 10:** Before compensation for strain, the improvement in the predicted results of XY relative to X and Y. (Unit: %).

Input	R^2^	MAE	MSE	RMSE
XY_X	20.21	−25.41	−37.56	−20.98
XY_Y	53.27	−29.17	−55.42	−33.23

**Table 11:** After compensation for strain, the improvement in the predicted results of XY relative to X and Y. (Unit: %).

Input	R^2^	MAE	MSE	RMSE
XY_X	18.18	−53.8	−67	−42.55
XY_Y	55.43	−64.26	−82.47	−58.13

**Table 12:** The improvement in the prediction results of each indicator after strain compensation compared to before strain compensation. (Unit: %).

Input	R^2^	MAE	MSE	RMSE
X	20.98	−25.63	−38.97	−21.88
Y	17.28	−8.704	−17.98	−9.435
XY	18.94	−53.94	−67.74	−43.20

**Table 13:** The setting status of the strain fault channels for the CT specimens.

Crack Length (mm)	5	15	25	35	45
X	*x*_3_	*x*_2_	*x*_1_	*x*_2_	*x*_3_
Y	*y*_1_	*y*_2_	*y*_3_	*y*_2_	*y*_1_

## Data Availability

Data are contained within the article.
